# miR172 Regulates *WUS* during Somatic Embryogenesis in Arabidopsis via *AP2*

**DOI:** 10.3390/cells11040718

**Published:** 2022-02-17

**Authors:** Katarzyna Nowak, Joanna Morończyk, Małgorzata Grzyb, Aleksandra Szczygieł-Sommer, Małgorzata D. Gaj

**Affiliations:** 1 Institute of Biology, Biotechnology and Environmental Protection, Faculty of Natural Sciences, University of Silesia, 40-007 Katowice, Poland; joanna.moronczyk@us.edu.pl (J.M.); aleksandra.sommer@gmail.com (A.S.-S.); malgorzata.gaj@us.edu.pl (M.D.G.); 2 Polish Academy of Sciences Botanical Garden—Center for Biological Diversity Conservation in Powsin, Prawdziwka 2, 02-973 Warsaw, Poland; m.grzyb@obpan.pl

**Keywords:** somatic embryogenesis, miR172, *AP2*, *WUS*, miR156, *SPLs*

## Abstract

In plants, the embryogenic transition of somatic cells requires the reprogramming of the cell transcriptome, which is under the control of genetic and epigenetic factors. Correspondingly, the extensive modulation of genes encoding transcription factors and miRNAs has been indicated as controlling the induction of somatic embryogenesis in Arabidopsis and other plants. Among the *MIRNA*s that have a differential expression during somatic embryogenesis, members of the *MIRNA172* gene family have been identified, which implies a role of miR172 in controlling the embryogenic transition in Arabidopsis. In the present study, we found a disturbed expression of both *MIRNA172* and candidate miR172-target genes, including *AP2*, *TOE1*, *TOE2*, *TOE3*, *SMZ* and *SNZ*, that negatively affected the embryogenic response of transgenic explants. Next, we examined the role of *AP2* in the miR172-mediated mechanism that controls the embryogenic response. We found some evidence that by controlling *AP2*, miR172 might repress the *WUS* that has an important function in embryogenic induction. We showed that the mechanism of the miR172-AP2-controlled repression of *WUS* involves histone acetylation. We observed the upregulation of the *WUS* transcripts in an embryogenic culture that was overexpressing *AP2* and treated with trichostatin A (TSA), which is an inhibitor of HDAC histone deacetylases. The increased expression of the *WUS* gene in the embryogenic culture of the *hdac* mutants further confirmed the role of histone acetylation in *WUS* control during somatic embryogenesis. A chromatin-immunoprecipitation analysis provided evidence about the contribution of HDA6/19-mediated histone deacetylation to AP2-controlled *WUS* repression during embryogenic induction. The upstream regulatory elements of the miR172-AP2-WUS pathway might involve the miR156-controlled SPL9/SPL10, which control the level of mature miR172 in an embryogenic culture.

## 1. Introduction

Somatic embryogenesis (SE), a plant-specific developmental process, results in the formation of embryos from in vitro-cultured somatic cells. For years, SE has been widely explored in plant biotechnology for the efficient regeneration, micropropagation and genetic modifications of plants [1]. Moreover, studies on SE have also provided an attractive research model for understanding the regulatory processes that control the embryogenic transition of somatic cells and, in a broader sense, the developmental plasticity and toti/pluripotency of plants [2].

The research of a model plant of Arabidopsis has contributed the most to revealing the molecular mechanisms that control SE induction [3]. The studies provided evidence that complex interactions between the genetic and epigenetic factors, including the transcription factors, miRNAs (microRNAs), DNA methylation and chromatin modifications, control the embryogenic reprogramming of somatic cells [4,5]. Consistent with a decisive function of the transcription factors (TFs) in the genetic reprogramming of somatic cells in plants and animals [6,7], hundreds of *TF* genes have shown an extensively modulated expression in the SE of different plants, including Arabidopsis [8,9,10,11,12,13]. Moreover, several TFs have been found to have an essential function in embryogenic transition, including *BABY BOOM*—*BBM* [14], *LEAFY* *COTYLEDON1* and *2*—*LEC1* and *LEC2* [15,16], *WUSCHEL*—*WUS* [17], *AGAMOUS-like15—AGL15* [18], *MYELOBLASTOSIS118*—*MYB118* [19] and *EMBRYOMAKER*—*EMK* [20]. Most SE-essential TFs control embryogenic induction by regulating the phytohormone-related pathways, mainly metabolism, transport and the signaling of auxin [3,5,21].

It is worth noting that the SE-essential TFs of Arabidopsis have been used to improve the regeneration efficiency of in vitro-recalcitrant crops [22,23,24,25]. This finding suggests that the SE-induction mechanism in different plants might have common regulators.

In concert with TFs, microRNAs control SE induction and a differential expression of numerous *MIRNA*s has been found in embryogenic cultures of Arabidopsis [26] and other plants [27,28,29]. Only a few miRNAs, including miR160 and miR166/165 [30], miR167 [31], miR393 [32], miR156 [33], miR396 [34] and miR528 [35], have been functionally analyzed during SE. The functions of miRNA in SE induction involve the regulation of the SE-involved genes. During SE, the miRNA-controlled TF-encoding genes regulate the genes that are involved in metabolism and the signaling of different phytohormones, mainly auxin [30,32,33,34,35], and the stress response [33,35].

The accumulation of the primary transcripts of *MIRNA172* genes in an embryogenic culture of Arabidopsis suggests SE-related functions of miR172 [26]. In vivo, miR172 controls the juvenile-to-adult phase transition and flowering in Arabidopsis [36,37]. Furthermore, the involvement of miR172 in regulating stem-cell fate [38], sex determination [36,39], fruit growth [40,41] and spike architecture [42] has been postulated. The function of miR172 in integrating various endogenous and exogenous cues during the developmental transitions that are associated with plant flowering has also been reported [43,44,45]. The regulatory mechanisms that control the development of the generative organs and the embryogenic transition of somatic cells seem to be partially convergent [8,11]. Thus, the role of miR172 in the genetic network controlling embryogenic induction in plant somatic cells might be hypothesized.

In Arabidopsis, five members (*MIR172a–e*) of a conserved *MIRNA172* gene family are spatially and temporally regulated and play redundant and specific roles in plant development [44,45,46]. Recently, the function of individual *MIRNA172* genes in regulating the flowering time in the leaves and the shoot apical meristem (SAM) was revealed [43]. The targets of miR172 include members of the AP2-like (APETALA-like) subfamily from the AP2/ERF (APETALA2/ETHYLENE RESPONSE FACTOR) family of TFs [47]. The AP2 protein family contains numerous members that are critical for SE in Arabidopsis and other plants, including *BBM* [14,48], the *PLETHORA* [34,49], *EMK* [20] and *Mt SOMATIC EMBRYO RELATED FACTOR1—MtSERF1* [50].

The miR172-controlled AP2-like genes include *AP2* (*APETALA2*), *TOE1* (*TARGET OF EAT1*), *TOE2*, *TOE3*, *SMZ* (*SCHLAFMÜTZE*) and *SNZ* (*SCHNARCHZAPFEN*), which regulate flowering and the development of the floral organs [37,51,52]. AP2-like TFs regulate the genes by the AP2-DNA-binding domain [47] and the targeted genes have been found to control the flower [53] and embryo development [14], spikelet meristem determinacy [54] and leaf epidermal-cell identity [55]. The AP2 targets during floral development include the floral repressor *AGL15* [56] and the stem-cell-niche regulator of the floral meristem, *WUS* [57]. Because *AGL15* and *WUS* have a regulatory function in embryogenic induction in vitro [17,18], the role of *AP2* in the regulatory circuit that controls SE could be assumed.

Besides the TFs and miRNAs, chromatin modifications, including histone acetylation (Hac), also control embryogenic development [58,59,60]. In Hac-mediated gene regulation, two types of enzymes, histone acetyltransferases (HATs) and histone deacetylases (HDACs), affect the accessibility of the chromatin-binding proteins to DNA via changes in the acetylation status of the histones [61]. The antagonist activity of HAT and HDAC promotes an open vs. closed chromatin state, which results in the activation vs. repression of gene transcription [62].

The central role of the epigenetic processes, including Hac in the SE-regulatory network, has been postulated [4]. However, the evidence on the Hac function during SE is still quite limited and mostly indirect. A substantial deregulation of the *HAT* and *HDAC* genes have been documented in SE induction in Arabidopsis [13] and other plants [63,64,65]. Moreover, the inhibition of HDAC by trichostatin A (TSA) treatment promoted the development of embryogenic structures in the seedlings and explants of Arabidopsis [66,67] and improved the embryogenic response in conifers [68,69]. The SE-promoting activity of TSA has been associated with the upregulation of *LEC1*, *LEC2*, *BBM* and *PHB* (*PHABOULOSA*), which implies a role of Hac in the regulation of the SE-involved *TF* genes [67]. However, the mechanisms and regulatory elements, including HDAC in the Hac-mediated regulation of specific genes in SE induction, remain mostly unexplored.

Of the HDACs, members of the RPD3/HDA1 (REDUCED POTASSIUM DEPENDENCE 3/HISTONE DEACETYLASE 1) family, including HDA6 and HDA19, have a regulatory function during various processes in plant development [70]. HDA6 and HDA19 redundantly control the repression of an embryo-specific gene function, and the repression of the HDA6/19 function results in a spontaneous somatic-embryo development in germinating seedlings [66], thereby suggesting the role of these HDACs during SE control. Recently, the cooperation of HDA6/HDA19 with the AGL15 and the TOPLESS co-repressors of the SIN3/HDAC (SWI-INDEPENDENT 3/HISTONE DEACETYLASE) silencing complex was revealed to control the miRNA biogenesis genes in SE induction [71].

The differential expression of the *MIRNA172* in the embryogenic culture of Arabidopsis [26], together with the key role of miR172 in the developmental transition of plants [44,45,46] motivated us to explore the function of miR172 in the embryogenic transition that is induced in in vitro-cultured explants of Arabidopsis. The goal of the study was to reveal the role of miR172 in the genetic network that controls SE induction by identifying the down- and upstream targets of miR172 in an embryogenic culture. The present study demonstrated that miR172 controls SE by targeting AP2, which represses the *WUS* expression. It also revealed that the miR172-AP2-imposed control of *WUS* in SE involves histone acetylation via the AP2-mediated recruitment of the histone deacetylases HDA6/HDA19 to the target gene. Moreover, it indicated upstream regulatory elements of the miR172-AP2-WUS module that involve miR156-controlled SPLs regulating the level of mature miR172 during SE. The results provided new components of the miRNA- and Hac-mediated pathways of the SE-regulatory network.

## 2. Materials and Methods

### 2.1. Plant Material and Growth Conditions

The Columbia (Col-0) seeds of *Arabidopsis thaliana* (L.) Heynh were supplied by NASC (The Nottingham Arabidopsis Stock Centre, Nottingham University, Nottingham, UK). The transgenic lines with a mutation in the *MIRNA172* (*miR172b*—N670873; *miR172c*—N673321; *miR172d*—N866316), miR172 target genes (*toe1*—N668454; *toe2*—N655709, *toe3*—CS825725, *smz*—N664087, *snz*—N668027, *ap2*—N571140) were purchased from the SALK Institute Genomic Analysis Laboratory database and the Syngenta Arabidopsis Insertion Library (SAIL). The 35S::*MIR172D* line was kindly provided by Dr. J. Palatnik (Research Council, Institute of Molecular and Cell Biology in Rosario, Argentina), 35S::MIM172 from Dr. Detlef Weigel (Max Planck Institute for Developmental Biology, Tübingen, Germany) and *hda6* and *hda6 hda19* by Kim Boutillier (Wageningen University & Research, Wageningen, The Netherlands). The 35S::*MIR172D* line had a higher level of mature miR172 molecules (Supplementary Figure S1). In the MIM lines (35S::MIM172, 35S::MIM156), the overexpressed transcripts with multiple miRNA-binding sites competed with the endogenous targets, thereby abolishing the function of all of the miRNA family members [72]. The 35S::*AP2*-ER and 35S::*SPL9*-ER lines with a β-estradiol-induced overexpression were purchased from the TRANSPLANTA collection [73]. In addition, the insertional mutants in *TOPLESS* (*tpl*—N68599, *tpr1*—N522964, *trp3*—N529936, *tpr4*—N502209), *SPLs* (*6mSPL10*—N66332, *6mSPL11*—N66336) and 35S::MIM156 (N9953) were studied. In the *6mSPL10* and *6mSPL11* lines, the mutation disrupted the miR156-binding site [74]. The plants that were used for the explants for the in vitro cultures were grown in Jiffy pots (Jiffy, Zwijndrecht, The Netherlands) in a “walk-in” type Phytotron under controlled conditions (20–22 °C, 16 h/8 h L/D photoperiod, light intensity of 100 μE m^−2^ s^−1^). The cultures that were grown in vitro were maintained in a controlled-growth chamber at 22 °C, 16 h/8 h (light/dark) and a light intensity of 40 µM m^−2^ s^−1^.

### 2.2. Somatic Embryogenesis Induction

Somatic embryogenesis was induced in vitro according to Gaj [75] and immature zygotic embryos (IZE) in the bent cotyledonary stage (10–12 DAP) were used as the explants. A solid B5-based [76] medium (E5) that had been supplemented with 20 g/L sucrose, 8 g/L Oxoid agar (Oxoid, Hampshire, UK) and 5 μM 2,4-D (2,4-dichlorophenoxyacetic acid, Sigma, St. Louis, MO, USA) was used to induce SE. In some of the experiments, the E5 medium was supplemented with a chemical inhibitor of the HDAC activity, trichostatin A (TSA; Sigma-Aldrich, St. Louis, MO, USA) at a concentration of 1 µM or β-estradiol (5 µM; Sigma-Aldrich, St. Louis, MO, USA) to induce an overexpression in the transgenic lines.

The morphogenic potential of the transgenic lines for SE was evaluated using two parameters: SE efficiency, which was calculated as the frequency of the explants that produced somatic embryos, and SE productivity, which was calculated as the average number of somatic embryos per explant. Thirty explants in three replicates were evaluated for each genotype.

### 2.3. Analysis of Mature miRNA and Target Genes Expression

Total RNA was isolated from the explants that had been induced on the E5, E5 + E, and E5 + TSA media for 0, 5 and 10 days. To isolate the RNA from the 0 d culture, IZEs that had been dissected from siliques in a drop of water were immediately transferred to 5 mL of RNAlater (Life Technologies, Carlsbad, CA, USA) and then treated the same way as the explants that that had been cultured for 5 and 10 d. Total RNA with miRNA were isolated using an miRVana miRNA Isolation Kit (Thermo Fisher Scientific, Waltham, MA, USA). Depending on the age of the culture, 250 (0 d) to 50 (10 d) explant-derived cultures were used for RNA isolation in one biological replicate. RNA concentrations were measured using a Nano-Drop ND-1000 (NanoDrop Technologies, Wilmington, DE, USA). The DNA was removed from the RNA samples by digesting them with RQ1 RNase-free DNase (Promega, Medison, WI, USA). The miRNA-specific and oligo-dT primers and a RevertAid First Strand cDNA synthesis kit (Thermo Fisher Scientific, Waltham, MA, USA) were used to produce the cDNA. The mature miRNAs were identified according to the Speth and Laubinger [77] method. The cDNA was diluted 1:4 and used for the Real-Time qPCR analysis. The qPCR was performed using a LightCycler^®^ 480 SYBR Green I Master kit (Roche, Basel, Switzerland) and the primers that were relevant to the genes being studied were used to determine the Real-Time RT qPCR reactions (Supplementary Table S1). The control gene had a constant expression level (C_T_ = 18 ± 1) in all of the tissue samples. The Ct values were calculated using LinRegPCR software (version 11, Academic Medical Centre, Amsterdam, The Netherlands). The plant tissues for the Real-Time qPCR analysis were produced in three biological repetitions and two technical replicates of each repetition were analyzed. The relative expression level was calculated using 2^−∆∆CT^, where ∆∆C_T_ represents ∆C_T_^reference condition^ − ∆C_T_ ^compared condition^.

### 2.4. Chromatin Immunoprecipitation (ChIP)

The ChIP method of Yelagandula et al. [78] with some modifications that were described by Nowak et al. [71] was used. Chromatin was extracted from the cultured explants (~50 mg of tissue) that had been treated with 1% formaldehyde for 20 min on ice under a vacuum. The chromatin was sheared using sonication (Bioruptor^®^ Plus, Diagenode, Denville, NJ, USA). The complex of proteins and DNA fragments was then immunoprecipitated using the polyclonal antibodies against the acetylated forms of histone H3 (2 µg; Merck, St. Louis, MO, USA, Cat. no. 06-599). The DNA that was cross-linked to the immunoprecipitated proteins was reversed and analyzed using qPCR and the gene-specific primers. A LightCycler 480 (Roche, Basel, Switzerland) real-time detection system was used to analyze the relative acetylation level of the *WUS*-gene-associated chromatin. The qPCR reaction was conducted according to Nowak et al. [71]. The primers that were used in the qPCR were designed using Primer3Plus software (version 3, Molbi, Michelstand, Germany). The genomic sequence that was analyzed in the *WUS* gene was localized in the TSS + 300 bp region and 3′UTR (Supplementary Table S1). The Ct values were calculated using LinRegPCR software (version 11, Academic Medical Centre, Amsterdam, The Netherlands). The ChIP–qPCR data were normalized using the percent-input method. The H3ac level is presented as 2^(adjusted input − Ct (xx gene)^ * 100%. Three biological replicates and two technical replicates were analyzed for each combination.

### 2.5. Statistical Analysis

The Student’s *t*-test and a two-way ANOVA (*p* < 0.05) followed by Tukey’s honestly-significant-difference test (Tukey HSD-test) (*p* < 0.05) were used to calculate any significant differences between the experimental combinations. The graphs show the average values with the standard deviation (SD); the statistical analysis was performed using the medians.

## 3. Results

### 3.1. Functional Analysis of the MIRNA172 Genes during SE

In a global analysis of pri-miRNA during SE, we found that five members of the *MIRNA172* gene family (*MIR172a-e*) were differentially expressed in the SE-induced explants of Arabidopsis [26].

**Figure 1:**
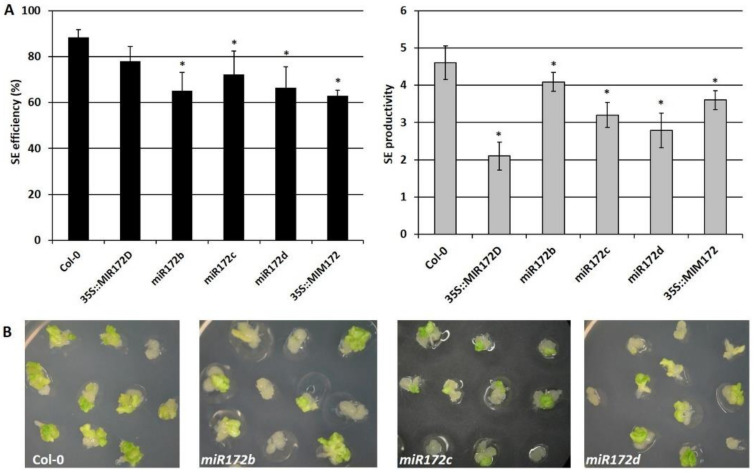
miR172 contributes to the embryogenic potential of a culture. There was a significant decrease in the SE efficiency and SE productivity of the miR172-affected genotypes, including the *miR172* mutants (*miR172b, miR172c, miR172d*), the *MIR172D*-overexpressing line (35S::*MIR172D*) and the MIM line (35S::MIM172) vs. the WT control (Col-0) culture (**A**). There was also a decrease in the embryogenic responses of the *miR172b*, *miR172c*, *miR172d* mutants compared to the Col-0 culture (**B**). The explants were induced on an auxin (E5) medium and the embryogenic response was evaluated in 21-day-old cultures. * values significantly different from the WT, Col-0 (*p* < 0.05; *n* = 3 ± SD).

To verify the hypothesis on the SE-related function of miR172, we analyzed the embryogenic potential of different lines with a disturbed expression/function of miR172, including T-DNA insertion mutants (*miR172b*, *miR172c*, *miR172d*), MIM line (35S::MIM172) and the overexpression line (35S::*MIR172D*). We found that all of the miR172-defective lines significantly impaired the SE response and both SE parameters, and that SE efficiency and SE productivity were reduced in the mutant cultures compared to the WT (wild type), Col-0 (Figure 1A,B).

The results indicate that a disturbed transcription of *MIR172* genes results in an impaired embryogenic response in the explants. The results also suggest that different *MIRNA* genes, including *MIR172b*, *c*, and *d* contribute to SE induction and that the tightly regulated miR172 level determines an efficient embryogenic response. Therefore, the contribution of miR172 to SE regulation might be postulated in Arabidopsis.

### 3.2. Targets of miR172 in SE Induction

**Figure 2:**
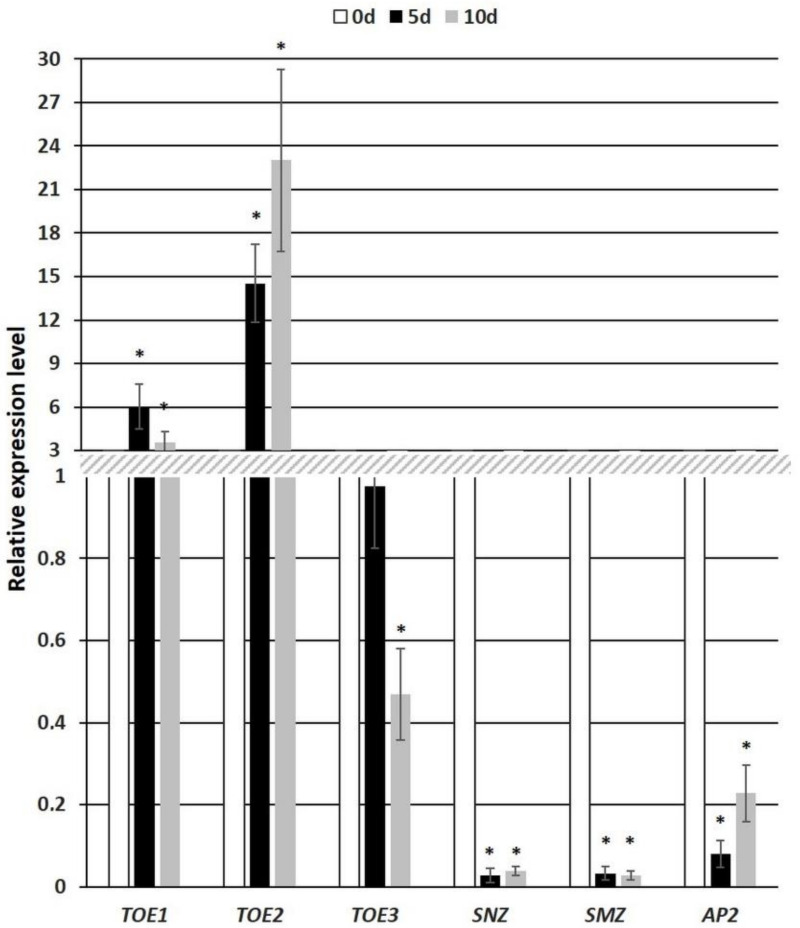
The expression level of the candidate miR172 targets, including *TOE1*, *TOE2*, *TOE3*, *SNZ*, *SMZ* and *AP2* in the embryogenic culture of Col-0. The relative transcript level was normalized to an internal control (*At4g27090*) and calibrated to 0 d of the culture. * values significantly different from the freshly isolated 0 d explants (*p* < 0.05; *n* = 3 ± SD).

The candidate miR172 targets include the transcription factors (TFs) that control plant flowering in vivo [79]. We used quantitative PCR to verify whether the expression of the miR172 targets is modulated during SE induction in the Col-0 genotype. An analysis of the candidates revealed a significant modulation of the *TOE1*, *2*, *3*, *SMZ*, *SNZ* and *AP2* transcripts and an increased *TOE1* and *TOE2* expression and decreased *SMZ*, *SNZ*, *AP2* and *TOE3* transcription in the SE-induced explants of Col-0 (Figure 2).

**Figure 3:**
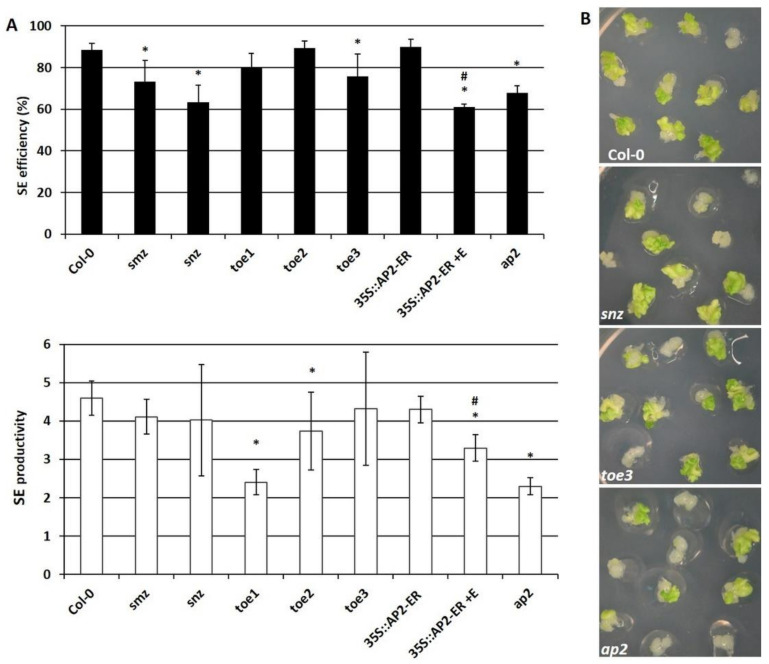
The impaired embryogenic response in the cultures affected the candidate miR172 target genes, including the *smz*, *snz*, *toe1*, *toe2*, *toe3* and *ap2* mutants and the *AP2*-induced overexpression line, 35S::*AP2*-ER, compared to the WT culture, Col-0. The significantly impaired SE efficiency and productivity (**A**) of the analyzed genotypes were exemplified by the reduced embryogenic response of the representative mutants *snz*, *toe3*, *ap2* (**B**). The explants were induced on an auxin (E5) medium and the embryogenic response was evaluated in 21-day-old cultures. The *AP2* overexpression was induced with β-estradiol (+E). * values significantly different from the WT, Col-0 (*p* < 0.05; *n* = 3 ± SD); #—values significantly different from the 35S::*AP2*-ER -E culture (*p* < 0.05; *n* = 3 ± SD).

To validate the involvement of the candidate miR172 targets in SE induction, we evaluated the embryogenic response of the *toe1*, *2*, *3*, *smz*, *snz* and *ap2* mutants and the 35S::*AP2*-ER-overexpression line. The results showed that all of the analyzed genotypes had a significantly defective embryogenic potential, which was manifested by a reduced SE efficiency and/or productivity (Figure 3A,B). The results support the assumption that the *TOE1*, *2*, *3*, *SMZ*, *SNZ* and *AP2* genes contribute to SE induction in Arabidopsis.

**Figure 4:**
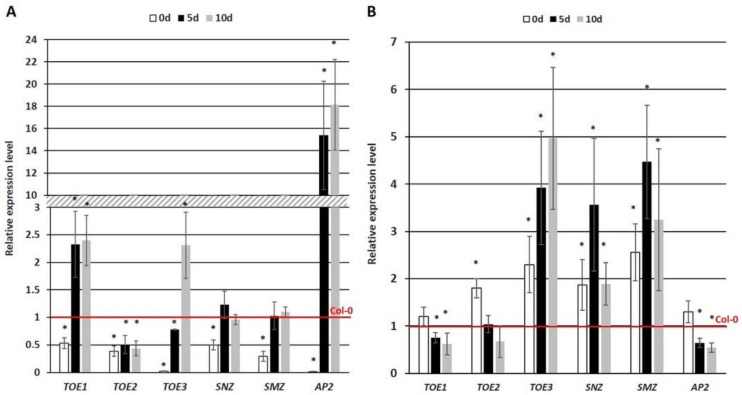
Expression analysis of the candidate miR172 target genes (*TOE1*, *TOE2*, *TOE3, SNZ*, *SMZ*, *AP2*) in the embryogenic cultures of the 35S::MIM172 (**A**) and 35S::*MIR172D* (**B**) lines with a disrupted miR172 function and an increased miR172 level, respectively. The relative transcript level was normalized to an internal control (*At4g27090*) and calibrated to the WT (Col-0) culture of the same age (0d, 5d and 10d). * values significantly different from the Col-0 culture of the same age (*p* < 0.05; *n* = 3 ± SD).

To further verify the regulatory relationship between miR172 and the candidate targets in SE induction, the transcription levels of *TOE1*, *2*, *3*, *SMZ*, *SNZ* and *AP2* were evaluated in the 35S::MIM172 and 35S::*MIR172D* cultures, which had a disrupted miR172 function and an increased miR172 level, respectively (Figure 4). The analysis showed that two candidate targets of miR172, *TOE1* and *AP2*, had contrasting expression levels in the 35S::MIM172 vs. the 35S::*MIR172D* cultures. Accordingly, the *TOE1* and *AP2* transcripts were substantially increased in the MIM172 (Figure 4A) and decreased in the 35S::*MIR172D* culture (Figure 4B). The results infer a role of miR172 in the control of *AP2* and *TOE1* in SE induction. In contrast to *TOE1* and *AP2,* the expression profiles of *TOE2, 3, SNZ* and *SMZ* in the 35S::MIM172 and 35S::*MIR172D* cultures were not indicative of their regulatory dependence on miR172 in SE. In plant development in vivo, the *WUS* TFs that have a regulatory function in SE was indicated among *AP2* targets [17,57,80]. Hence, we focused our further analysis on the role of the miR172-AP2-WUS pathway in controlling the embryogenic response.

### 3.3. miR172 Regulates WUS TF of Critical Function during SE via AP2

**Figure 5:**
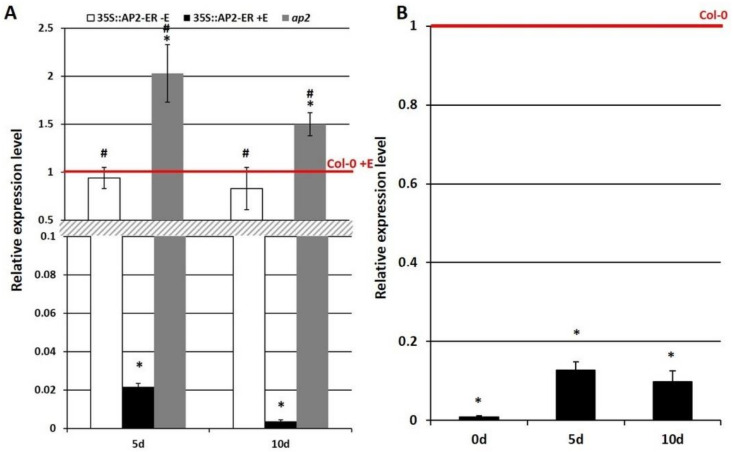
Expression analysis of *WUS* in the SE culture in *AP2*-induced overexpression line and *ap2* mutant (**A**) and 35S::MIM172 line with a disrupted miR172 function (**B**). *AP2* overexpression was induced with β-estradiol (+E). The relative transcript level was normalized to an internal control (*At4g27090*) and calibrated to the WT (Col-0) culture of the same age (0d, 5d and 10d). * values significantly different from the Col-0 culture of the same age (*p* < 0.05; *n* = 3 ± SD); #—values significantly different from the 35S::*AP2*-ER +E culture of the same age (*p* < 0.05; *n* = 3 ± SD).

To gain insight into the regulatory relationship between *AP2* and *WUS* in SE induction, the expression of *WUS* was analyzed in the SE-induced explants with an increased (35S::*AP2*-ER) or defective (*ap2*) *AP2* activity. We found that the level of the *WUS* transcript was decreased and increased in the SE-explants of increased (35S::*AP2*-ER) and an impaired (*ap2*) *AP2* expression, respectively, which suggests that AP2 negatively regulates *WUS* in SE induction (Figure 5A). Because we had assumed that *AP2* would be under the control of miR172 during SE (Figure 4), we examined whether disrupting the miR172 activity would affect the expression level of the candidate AP2 target, *WUS*. The results showed a decrease in the *WUS* transcripts in the 35S::MIM172 culture, which suggests a regulatory relationship between miR172 and *WUS* during SE (Figure 5B).

To summarize, the results provided evidence that miR172 might indirectly regulate the expression of the *WUS* TFs that play a critical role in SE induction by controlling *AP2*.

### 3.4. AP2 Represses the WUS Expression via a HDAC-Mediated Histone Acetylation

**Figure 6:**
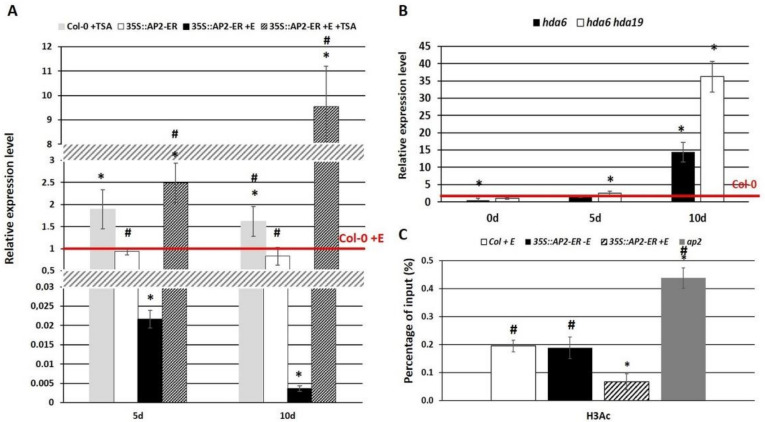
A Hac-related mechanism is involved in the AP2-mediated repression of *WUS* during SE. Expression analysis of *WUS* in the SE culture in the *AP2*-overexpression line untreated (-TSA) and the one that had been treated (+TSA) with TSA (trichostatin A) (**A**) and the *hda6* and *hda6 hda1*9 mutants (**B**). Hac enrichment in the chromatin fragment that was bound to the TSS + 300 bp region of the *WUS* gene promoter in the explants of Col-0 (WT), 35S::*AP2*-ER and *ap2* (**C**). *AP2* overexpression was induced with β-estradiol (+E). The HDAC activity was inhibited by the TSA treatment in the Col-0 control (Col-0 +TSA) and in the *AP2*-overexpressing (35S::*AP2*-ER +TSA) culture. The relative transcript level was normalized to an internal control (*At4g27090*) and calibrated to the WT (Col-0) culture of the same age (0d, 5d, and 10d). * values significantly different from the Col-0 culture of the same age (*p* < 0.05; *n* = 3 ± SD); #—values significantly different from the 35S::*AP2*-ER +E culture of the same age (*p* < 0.05; *n* = 3 ± SD).

We assumed that the AP2-mediated repression of *WUS* in SE induction might involve a HDAC-controlled histone deacetylation. It is known that histone acetylation is involved in the AP2-mediated mechanism of target-gene repression during plant development [81,82]. To verify whether Hac contributes to the AP2-controlled *WUS* expression during SE, we analyzed the *WUS* transcription level in the explants that overexpressed *AP2* and those that had been treated with an inhibitor of HDAC, trichostatin A (TSA), to block the HDAC function. The TSA-induced explants had an accumulation of the *WUS* transcript in response to an *AP2* overexpression (Figure 6A). A similar tendency was observed for the Col-0 culture that had been treated with TSA. The results suggest that a repressive activity of AP2 on *WUS* expression might be exerted via the HDAC-related pathway. The candidate HDAC with an assumed role in controlling the SE-involved genes include the HDA6 and HDA19 histone deacetylases [66,71]. Our analysis of the *WUS* expression relative to the *HDA6/HDA19* expression revealed an elevated *WUS* transcript in the SE-induced explants of the *hda6* and *hda6 hda19* mutants (Figure 6B). The results imply that AP2 might repress the expression of the *WUS* gene during SE via a HDA6/HDA19-mediated histone deacetylation. To find further evidence for the assumption that Hac is involved in the AP2-mediated regulation of the *WUS* expression, we examined changes in the H3 histone acetylation marks (H3K9ac and H3K14ac) in the chromatin that are associated with the TSS + 300 bp fragment of the *WUS* relative to the *AP2* expression. The results of the ChIP analysis indicated that the level of H3 acetylation in the *WUS*-bound chromatin region (TSS +300 bp) was increased in the *ap2* mutant and decreased in the *AP2* overexpressing cultures, respectively (Figure 6C). In contrast to the TSS +300bp, the chromatin fragment that was bound to the 3′UTR of *WUS* had a similar H3ac level in the cultures of Col-0, the *AP2*-overexpression line and the *ap2* mutant (Supplementary Figure S2). To summarize, we documented that AP2 might negatively control the *WUS* gene in SE induction via a Hac-related mechanism in which HDA6 and HDA19 seem to be involved.

### 3.5. TPL Co-Repressors Might Contribute to the AP2-Mediated Repression of WUS during Embryogenic Induction

**Figure 7:**
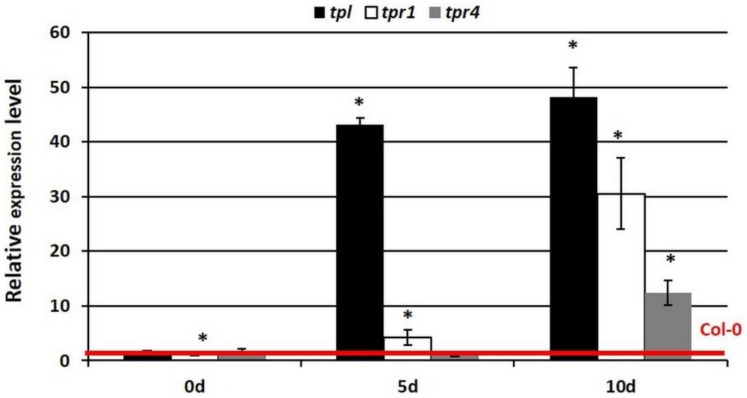
Expression analysis of *WUS* in the SE culture in the TOPLESS co-repressor mutants—*tpl*, *tpr1* and *tpr4* mutants. The relative transcript level was normalized to an internal control (*At4g27090*) and calibrated to the WT (Col-0) culture of the same age (0d, 5d, and 10d). * values significantly different from the Col-0 culture of the same age (*p* < 0.05; *n* = 3 ± SD).

Next, we hypothesized that the TOPLESS (TPL) co-repressors that directly interact with AP2 during plant development [81] would also be involved in the AP2-mediated repression of the *WUS* gene during SE. To verify this assumption, we evaluated the expression level of *WUS* in the *tpl/tpr* mutant cultures and compared it to the Col-0 control of the same age. The analysis showed that the *WUS* gene was significantly up-regulated in the SE-induced explants of the *tpl*, *tpr1* and *tpr4* mutants (Figure 7). The results suggest a contribution of the TPL (TPL, TPR1 and TPR4) co-repressors to the AP2-mediated negative control of *WUS* in SE induction.

### 3.6. miR156 Might Control the miR172-AP Regulatory Module during SE by Targeting SPL9/10

**Figure 8:**
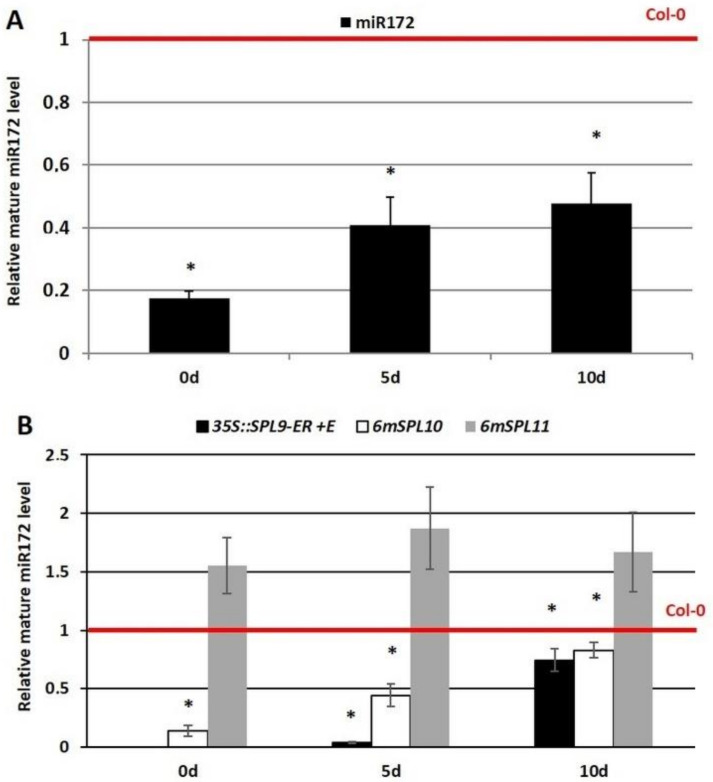
Level of mature miR172 in the embryogenic culture of 35S::MIM156 with a disrupted miR156 function (**A**) and an *SPL9*-overexpression line (35S::*SPL9*-ER) and the *6mSPL10* and *6mSPL11* mutants with a disrupted miR156-binding site (**B**). *SPL9* overexpression was induced with β-estradiol (+E). The relative miRNA level was normalized to an internal control (*At4g27090*) and calibrated to the WT (Col-0) culture of the same age (0d, 5d, and 10d). *—value significantly different from the Col-0 culture of the same age (*p* < 0.05; *n* = 3 ± SD).

Our results provide some evidence that miR172 might control the *WUS* gene in SE induction by repressing *AP2*. Next, we attempted to identify the upstream regulators of the miR172-AP2-WUS regulatory module. The candidate regulators included the miR156-repressed SPL (SQUAMOSA-PROMOTER BINDING PROTEIN-like) TFs that control the miR172-AP2 module during the floral transition [45,51,83]. Therefore, to verify the regulatory relationship between the miR156- and miR172-controlled modules in SE induction, we evaluated the accumulation of miR172 in the SE-induced explants with an abolished miR156 function (35S::MIM156) (Figure 8A) and an overexpression of three of the *SPL* genes, *SPL9/SPL10/SPL11* in the culture of 35S::*SPL9-ER*, *6mSPL10* and *6mSPL11* lines (Figure 8B). The results showed a decrease in the mature miR172 levels in 35S::MIM156 and *SPL9/10*-overexpressing lines (Figure 8). Thus, the results imply that miR156 might negatively control miR172 in embryogenic induction in Arabidopsis by targeting *SPL9* and *10*.

**Figure 9:**
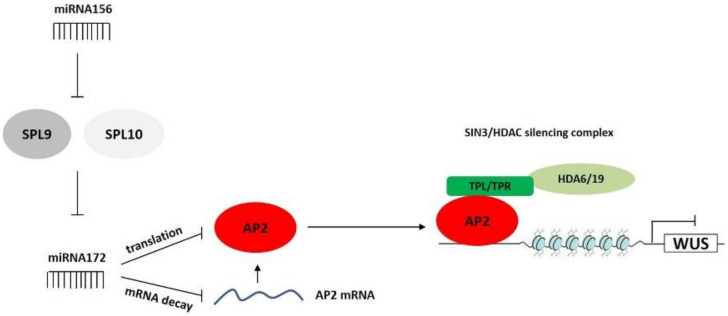
A putative model of the miR172-mediated regulatory interactions that control SE induction by repressing AP2 controls the *WUS* TF with a regulatory function in embryogenic induction. The AP2-controlled repression of *WUS* involves a Hac-related mechanism in which AP2 might recruit TOPLESS co-repressors (TPL, TPR1, TPR4) and histone deacetylases, HDA6 and HDA19, which are the central components of the SIN3/HDAC gene-silencing complex. Other miR172-related regulatory elements that might regulate SE induction include the miR156-SPL9/10 module, which has a negative impact on miR172.

To summarize, the study provides several pieces of evidence on new, miR172-related regulatory components of the genetic network that underlies SE induction. In this mechanism, miR172-controlled AP2 in cooperation with the TPL co-repressors and HDA6/HDA19 negatively controls *WUS* during SE. The upstream-acting elements that control the miR172-AP2 module during SE might also involve the miR156-SPL regulatory module (Figure 9).

## 4. Discussion

### 4.1. miR172 Controls SE Induction by Targeting AP2

The genetic network that controls the SE induction involves complex regulatory interactions in which miRNA-mediated gene repression plays a substantial role [4]. Consistent with this notion, the differential expression of numerous pri-miRNAs of different *MIRNA* gene families, including *MIRNA172*, was found in SE-induced explants of Arabidopsis [26]. Like Arabidopsis, a differential transcription and accumulation of *MIRNA172* and mature miR172, respectively, have also been associated with SE induction in other plants, thus further ensuring the role of miR172 in SE regulation [27,28,84,85,86]. However, the miR172-mediated regulatory mechanism, including the up- and downstream targets of miR172 in the embryogenic induction, is as yet unknown.

Therefore, we conducted a functional analysis of miR172 in the SE of Arabidopsis. The results showed that both an overexpression (35S::*MIR172D*) and a disturbed function (35S::MIM172) of miR172 resulted in an impaired embryogenic response of the explants (Figure 1). Congruently, the genotypes that were affected in *AP2*, which is the miR172 target, including the *ap2* mutant and *AP2*-overexpression line, also had a reduced SE induction (Figure 3). The impaired SE response was also characteristic of the genotypes with an increased or decreased expression/activity of other SE-regulators, including *LEC2*, *ARF5* (*AUXIN RESPONSE FACTOR5*), and miR393 [16,32,87,88]. Thus, we assume that a strictly controlled level of miR172 and *AP2* seems to be required for efficient SE induction.

Two alternative modes of miR172-mediated gene regulation, cleavage and a translation inhibition of the target mRNA, have been suggested [89,90,91,92]. The opposite level of miR172 vs. the *AP2* and *TOE1* transcripts (Figure 4) suggests that miR172 might control these genes in SE at the transcriptional level via mRNA cleavage. The regulatory role of the miR172-AP2 module in the developmental transition that is associated with plant flowering [80] and a postulated similarity of the flower regulators to that controlling the embryogenic transition of somatic cells [12,48] motivated us to investigate the downstream target of the AP2 TF during SE. The results of the gene-expression profiling in the *miR172*-affected cultures (Figure 4) suggests that unlike *AP2* and *TOE1*, other potential targets, including *TOE2*, *3*, *SNZ* and *SMZ*, seem not to be regulatory dependent on miR172 in SE. In contrast to SE, miR172 controls these genes during plant flowering and the development of the floral organs [37,51,52]. The report and the present results imply that the targets of miR172 differ between the developmental processes. In line with the distinctly different expression profiles of the members of the *MIRNA172* gene family in plant developmental processes, including SE [26,44,45,93,94], the regulatory relations between miR172 and the targets are highly specific to the developmental context. In support of this, miR172 controls the *AP2* gene in flower development [93], whereas it targets *TOE1* and *TOE2* to promote juvenile epidermal identity in the production of the trichomes [51]. It is worth noting that the two closest *AP2* homologues, *TOE3* and *TOE1*, do not act redundantly to AP2 in stem-cell maintenance [57]. In the control of flowering time, *SMZ*, *SNZ*, *TOE1* and *TOE2* are involved but not *TOE3* [46].

### 4.2. The AP2 Module Controls WUS in Embryogenic Induction via a Hac-Related Repression

In the shoot apical meristem, AP2 regulates *WUS* in order to control the WUS-CLV3 (CLAVATTA3) feedback loop that has an essential function in stem-cell maintenance [57]. WUS, a member of the plant-specific homeobox superfamily of WOX TFs, has also been implicated in controlling auxin-induced SE [17]. The early induction of *WUS* expression is critical for embryo development in the embryogenic callus of Arabidopsis [95]. The interaction of WUS with the LEC-controlled pathway of SE induction has also been postulated [96]. However, the upstream regulators that control the auxin-gradient-induced *WUS* expression in SE have not yet been identified.

We provide some evidence that AP2 might repress *WUS* expression in controlling the embryogenic transition in Arabidopsis. In support of this, we found that there was an opposite effect of the *ap2* mutation vs. *AP2* overexpression of *WUS* expression during SE (Figure 5). The presence of the AP2-recognized *cis*-element in the *WUS* promotor suggests that AP2 might directly control *WUS* transcription. Further analysis is required to verify the role of the AP2-WUS interactions in regulating *WUS* expression during SE.

We found histone-acetylation-related mechanisms of the AP2-mediated repression of the *WUS* gene in SE induction. In support of this, the repressive effect of AP2 on the *WUS* transcripts was abolished in cultures that had been treated with TSA, which is an HDAC inhibitor (Figure 6). In addition, the *AP2* expression negatively affected the H3ac level in the *WUS* TSS + 300 region in the SE-induced explants.

Both activating and repressive functions of AP2 in controlling gene expression have been documented [80]. AP2 interacts with the SIN3/HDAC-silencing-complex components, including the transcriptional co-repressors and HDAC, in order to repress the target genes [81,82]. The histone deacetylases that cooperate with AP2 include HDA6 and HDA19 [82]. Our findings of an increased *WUS* expression in the SE-induced *hda6* and *hda6* *had19* explants (Figure 6) support the assumption of the role of HDA6 and HDA19 in the AP2-mediated repression of the *WUS* gene during embryogenic transition.

In the gene-repression mechanism, TFs cooperate with the transcriptional co-repressors, and in order to repress its targets, AP2 recruits the TOPLESS co-repressors into the SIN3/HDAC-silencing complex [81,82]. The results imply a role of TPL, TPR1 and TPR4 in the AP2-mediated repression of the *WUS* gene in an embryogenic culture (Figure 7). Accordingly, the *tpl/tpr* mutant cultures had a higher *WUS* expression. Two of these co-repressors, TPL and TPR1, can physically interact with AP2 via the EAR (Ethylene-responsive element-binding factor that is associated with amphiphilic repression) domain [81], but the character of the interactions between AP2-TPL/TPR1 needs to be verified during SE.

The importance of gene repression in plant development, including SE induction, is increasingly being recognized [97,98,99]. However, identifying the regulatory components of the gene-silencing mechanisms during SE has just begun. Recently, the SIN3/HDAC-silencing complex was reported to transcriptionally control the genes that are involved in the miRNA biogenesis during SE [71]. In this mechanism, AGL15 recruits the TPL/TPR co-repressors and cooperates with HDA6/HDA19 in order to repress the targets, *DCL1* (*DICER-like3*), *SERRATE* and *HEN1* (*HUA-ENHACER1*) [71]. We postulated that a similar silencing complex might repress *WUS* transcription in SE induction. To silence the *WUS* in SE, AP2 TF might recruit the TPL, TPR1 and TPR4 co-repressors and HDA6/HDA19 into the SIN3/HDAC complex.

TPL/TPRs might also physically interact with numerous other TFs, including members of the AUX/IAA, MYB, NAC and MADS gene families that have been indicated/suggested as being involved in SE induction [8,12,13,17,100,101]. Thus, we assumed that some of these TFs might have a regulatory function in SE induction as a part of the SIN3/HDAC complex. Identifying the TFs that contribute to the SIN3/HDAC-complex-mediated regulation seems to be interesting in order to further reveal the SE-regulatory pathways.

### 4.3. miR156-Targeted SPL9 and SPL10 Negatively Regulate the miR172 Level during SE

The study revealed the role of the miR172-AP2 module in regulating *WUS* in SE induction. During plant development, the upstream regulators of miR172-AP2 and other miR172-AP2-like modules, include the miR156-SPLs [46]. The regulatory functions of the miR156-SPLs in the fundamental developmental processes have been documented [102], including the involvement of the miR156 and *SPL10* and *11* genes in zygotic-embryo development [74]. We assumed that the miR156-SPL module might also control SE. In support of this hypothesis was the opposite expression levels of the mature miR156 vs. the *SPL9*, *10* and *11* transcripts that have been reported in an embryogenic culture of Arabidopsis [26]. Moreover, an analysis of transgenic lines in a callus culture of citrus provided some evidence that miR156 enhanced the SE induction by silencing *SPL3* and *SPL14* [33].

During the floral transition, the miR156-regulated SPLs, including SPL9/10 and 15, positively affect the expression of the *MIRNA172* genes [45,51,103]. Our analysis of the Arabidopsis explants that overexpressed components of the miR156-SPL module indicated that the *SPL9* and *SPL10* negatively control the amount of miR172 in SE induction (Figure 8).

The SPL-mediated regulatory mechanisms involve the positive control of targets by direct binding of SPLs to target promoters and competitive interactions with other regulatory proteins, including TCP4 (TEOSINTE BRANCHED 1 CYCLOIDEA, PCF1 FAMILY TRANSCRIPTION FACTOR 4) and the CUC (CUP-SHAPED) TFs, in the transcriptional complexes [104,105]. On the other hand, the SPL-mediated repression of gene transcription has also been documented. In support of this, SPL9 negatively controlled the *LAS* (*LATERAL SUPPRESSOR*), which is a regulator in the formation of the axillary bud in Arabidopsis [106].

The presence of the EAR repression domain whose role is to recruit HDAC into the silencing complex [107,108] in some of the SPLs (PlantEAR database- [109]) provides a hint that Hac might be involved in SPL-controlled repression, including the negative control of miR172 by SPL10 during SE.

The assumed mechanism by which SPL9, which lacks the EAR domain, represses miR172 during SE might involve SPL9 interactions with the components of the phytohormone-signaling pathways such as gibberellin (GA) and strigolactone (SL) [110,111]. Given the central function of the phytohormones, including GA, in controlling the SE-involved genes, the contribution of the DELLA and D53-like regulators of GA and SL signaling pathways, respectively, to the SPL9-mediated repression of miR172 might also be of interest in future studies on SE [112].

To summarize, we provide some evidence that miR156-regulated SPL9/SPL10 [26] might control the miR172-AP2 node in order to repress *WUS* expression in SE induction in Arabidopsis. Similarly, the direct and indirect impact of the miR156-SPLs on *WUS* in controlling the SAM size has also been reported [113]. Moreover, the physical interactions of the SPL9 and WUS proteins that have been found for some soybean orthologues suggest that the SPL proteins might also post-translationally regulate WUS activity [114]. Further analyses are required in order to verify the regulatory interactions between the components of the miR156-SPLs-miR172-AP2-WUS during SE.

### 4.4. Other Candidate Components of the miR172-Mediated Pathways in SE, AGL15 and TOE1

The versatile regulatory interactions of the miR172-AP2 module during plant development [46] suggest that besides *WUS*, other genes might be targeted by AP2 during SE. Insight into the genes that are controlled by miR172-AP2 during the floral transition [115] that have a postulated regulatory similarity to SE induction [8,11,12] revealed that AGL15 plays a role during SE [18]. AGL15 contributes to SE induction by controlling the genes involved in the hormone and stress responses and miRNA biogenesis [71,116,117,118]. Regulatory interactions between AGL15 and other SE-involved TFs, including the *LEC* genes, have also been documented [3]. However, little is known about the upstream elements that control the *AGL15* expression during the embryogenic response. We assume that AP2 might activate the *AGL15* expression during SE as we found a positive impact of AP2 on *AGL15* expression (Supplementary Figure S3). The role of AP2-AGL15 regulatory interactions and the relevance of the *AGL15* expression to miR172 during SE remain to be explored in future research.

Our results suggest that, besides AP2, miR172 might repress another AP2-like TF, *TOE1*, during SE. The hypothesis on the TOE1 function in SE induction is of particular interest given the interactions of TOE1 with KANADI1 (KAN1), which is the regulator of the development of the abaxial trichome of leaves [119]. Like leaves, the cotyledons have an adaxial–abaxial polarity [120] and the adaxial side of cotyledon exclusively contributes to somatic-embryo development in Arabidopsis [121,122]. In support of the role of the cotyledon polarity in the SE-induction mechanism, miR165/miR166 and *PHB* had adaxial-specific expression patterns in the SE-induced explants [30,123]. Importantly, during SE, to control the abaxial leaf identity, KAN1 targets the genes that are engaged in auxin biosynthesis, auxin transport and auxin response [124], which play central roles in the SE-induction mechanism [3]. Therefore, the TOE1 function relative to KAN1 and the adaxial-specific SE induction needs to be addressed in future research on the miR172-mediated control during SE.

## 5. Conclusions

The coordinated actions between the miRNAs and epigenetic modifications are believed to significantly contribute to regulating gene expression during developmental processes [35,125,126]. The present study suggests a contribution of miRNAs and Hac to the regulation of *WUS* during the embryogenic response of Arabidopsis explants. We provide some evidence that the miR172-AP2 regulatory node under the control of the miR156-SPL module might repress *WUS* expression in SE induction in Arabidopsis via a Hac-related mechanism. In this mechanism, the cooperation of the AP2 TF with the TPL/TPR1/TPR4 co-repressors results in the recruitment of HDACs such as HDA6/HDA19 into the SIN3/HDAC-silencing complex.

Other candidates in the HDAC-mediated regulation include hundreds of TFs that have the EAR repression domain [109]. Within these, the transcriptional repressors VAL1 and VAL2, which have been indicated as playing a role in SE induction [127], seem to be of particular interest in studies on epigenetics, including a Hac-mediated gene regulation during SE. VAL1/VAL2 recruit the histone-methylation-related Polycomb Repressive Complex 2 (PRC2) into gene silencing in Arabidopsis [128]. Interestingly, VAL1 has recently been found to couple the PRC2-controlled histone methylation with HDAC in the mechanism of gene repression [129]. Thus, we assume that the *VAL* genes would be of particular interest in studies on the embryogenic reprogramming of the plant somatic cells and, in particular, on the interplay of Hac with other epigenetic modifications such as histone methylation.

## Data Availability

Not applicable.

## Supplementary Materials

The following are available online at https://www.mdpi.com/article/10.3390/cells11040718/s1, Figure S1. Level of mature miR172 in the embryogenic culture and 14-day-old seedlings of the 35S::*MIR172D* line. The relative miRNA level was normalized to an internal control (*At4g27090*) and calibrated to the WT (Col-0) culture of the same age (0d, 5d, and 10d) and 14-day-old seedlings. *—value significantly different from the Col-0 culture of the same age (*p* < 0.05; *n* = 3 ± SD) and 14-day-old seedlings. Table S1: List of the primers that were used for the Real Time qPCR analysis. Figure S2. H3ac enrichment in the chromatin fragment that was bound to the 3′UTR of the *WUS* gene in the explants of Col-0 (WT), 35S::*AP2*-ER and *ap2*. *AP2* overexpression was induced with β-estradiol (+E). A statistical analysis indicated no differences between the compared combinations (*p* < 0.05; *n* = 3 ± SD). Figure S3: Expression analysis of *AGL15* in the SE culture in *AP2*-induced overexpressor line and the *ap2* mutant. The relative transcript level was normalized to an internal control (*At4g27090*) and calibrated to the WT (Col-0) culture of the same age. * values significantly different from the Col-0 culture of the same age (*p* < 0.05; *n* = 3 ± SD); #—values significantly different from the 35S::*AP2*-ER -E culture of the same age (*p* < 0.05; *n* = 3 ± SD).

## References

[1] Ochoa-Alejo N., Loyola-Vargas V.M., Ochoa-Alejo N. (2016). The uses of somatic embryogenesis for genetic transformation. Somatic Embryogenesis: Fundamental Aspects and Applications.

[2] Costa S., Shaw P. (2007). “Open minded” cells: How cells can change fate. Trends Cell Biol..

[3] Wójcik A.M., Wójcikowska B., Gaj M.D. (2020). Current perspectives on the auxin-mediated genetic network that controls the induction of somatic embryogenesis in plants. Int. J. Mol. Sci..

[4] Wójcikowska B., Wójcik A.M., Gaj M.D. (2020). Epigenetic regulation of auxin-induced somatic embryogenesis in plants. Int. J. Mol. Sci..

[5] Salaün C., Lepiniec L., Dubreucq B. (2021). Genetic and molecular control of somatic embryogenesis. Plants.

[6] Jaenisch R., Young R. (2008). Stem cells, the molecular circuitry of pluripotency and nuclear reprogramming. Cell.

[7] Su Y.H., Tang L.P., Zhao X.Y., Zhang X. (2021). Plant cell totipotency: Insight into cellular reprogramming. J. Integr. Agric..

[8] Thibaud-Nissen F., Shealy R.T., Khanna A., Vodkin L.O. (2003). Clustering of microarray data reveals transcript patterns associated with somatic embryogenesis in soybean. Plant Physiol..

[9] Che P., Love T.M., Frame B.R., Wang K., Carriquiry A.L., Howell S.H. (2006). Gene expression patterns during somatic embryo development and germination in maize Hi II callus cultures. Plant Mol. Biol..

[10] Hosp J., Tashpulatov A., Roessner U., Barsova E., Katholnigg H., Steinborn R., Melikant B., Lukyanov S., Heberle-Bors E., Touraev A. (2007). Transcriptional and metabolic profiles of stress-induced, embryogenic tobacco microspores. Plant Mol. Biol..

[11] Sharma S.K., Millam S., Hedley P.E., McNicol J., Bryan G.J. (2008). Molecular regulation of somatic embryogenesis in potato: An auxin led perspective. Plant Mol. Biol..

[12] Gliwicka M., Nowak K., Balazadeh S., Mueller-Roeber B., Gaj M.D. (2013). Extensive modulation of the transcription factor transcriptome during somatic embryogenesis in *Arabidopsis thaliana*. PLoS ONE.

[13] Wickramasuriya A.M., Dunwell J.M. (2015). Global scale transcriptome analysis of Arabidopsis embryogenesis in vitro. BMC Genomics.

[14] Boutilier K., Offringa R., Sharma V.K., Kieft H., Ouellet T., Zhang L., Hattori J., Liu C.M., Van Lammeren A.A.M., Miki B.L.A. (2002). Ectopic expression of BABY BOOM triggers a conversion from vegetative to embryonic growth. Plant Cell.

[15] Lotan T., Ohto M.A., Matsudaira Yee K., West M.A.L., Lo R., Kwong R.W., Yamagishi K., Fischer R.L., Goldberg R.B., Harada J.J. (1998). Arabidopsis LEAFY COTYLEDON1 is sufficient to induce embryo development in vegetative cells. Cell.

[16] Gaj M.D., Zhang S., Harada J.J., Lemaux P.G. (2005). Leafy cotyledon genes are essential for induction of somatic embryogenesis of Arabidopsis. Planta.

[17] Zuo J., Niu Q.-W., Frugis G., Chua N.-H. (2002). The WUSCHEL gene promotes vegetative-to-embryonic transition in Arabidopsis. Plant J..

[18] Harding E.W., Tang W., Nichols K.W., Fernandez D.E., Perry S.E. (2003). Expression and maintenance of embryogenic potential is enhanced through constitutive expression of AGAMOUS-Like 15. Plant Physiol..

[19] Wang X., Niu Q.W., Teng C., Li C., Mu J., Chua N.H., Zuo J. (2009). Overexpression of PGA37/MYB118 and MYB115 promotes vegetative-to-embryonic transition in Arabidopsis. Cell Res..

[20] Tsuwamoto R., Yokoi S., Takahata Y. (2010). Arabidopsis EMBRYOMAKER encoding an AP2 domain transcription factor plays a key role in developmental change from vegetative to embryonic phase. Plant Mol. Biol..

[21] Horstman A., Li M., Heidmann I., Weemen M., Chen B., Muino J.M., Angenent G.C., Boutiliera K. (2017). The BABY BOOM transcription factor activates the LEC1-ABI3-FUS3-LEC2 network to induce somatic embryogenesis. Plant Physiol..

[22] Deng W., Luo K., Li Z., Yang Y. (2009). A novel method for induction of plant regeneration via somatic embryogenesis. Plant Sci..

[23] Heidmann I., de Lange B., Lambalk J., Angenent G.C., Boutilier K. (2011). Efficient sweet pepper transformation mediated by the BABY BOOM transcription factor. Plant Cell Rep..

[24] Belide S., Zhou X.R., Kennedy Y., Lester G., Shrestha P., Petrie J.R., Singh S.P. (2013). Rapid expression and validation of seed-specific constructs in transgenic LEC2 induced somatic embryos of Brassica napus. Plant Cell. Tissue Organ Cult..

[25] Lowe K., Wu E., Wang N., Hoerster G., Hastings C., Cho M.-J., Scelonge C., Lenderts B., Chamberlin M., Cushatt J. (2016). Morphogenic Regulators Baby boom and Wuschel Improve Monocot Transformation. Plant Cell.

[26] Szyrajew K., Bielewicz D., Dolata J., Wójcik A.M., Nowak K., Szczygieł-Sommer A., Szweykowska-Kulinska Z., Jarmolowski A., Gaj M.D. (2017). MicroRNAs are intensively regulated during induction of somatic embryogenesis in Arabidopsis. Front. Plant Sci..

[27] Zhang S., Zhou J., Han S., Yang W., Li W., Wei H., Li X., Qi L. (2010). Four abiotic stress-induced miRNA families differentially regulated in the embryogenic and non-embryogenic callus tissues of Larix leptolepis. Biochem. Biophys. Res. Commun..

[28] Yang X., Wang L., Yuan D., Lindsey K., Zhang X. (2013). Small RNA and degradome sequencing reveal complex miRNA regulation during cotton somatic embryogenesis. J. Exp. Bot..

[29] Chávez-Hernández E.C., Alejandri-Ramírez N.D., Juárez-González V.T., Dinkova T.D. (2015). Maize miRNA and target regulation in response to hormone depletion and light exposure during somatic embryogenesis. Front. Plant Sci..

[30] Wójcik A.M., Nodine M.D., Gaj M.D. (2017). MiR160 and miR166/165 contribute to the LEC2-mediated auxin response involved in the somatic embryogenesis induction in arabidopsis. Front. Plant Sci..

[31] Wójcik A.M., Mosiolek M., Karcz J., Nodine M.D., Gaj M.D., Jenik P.D., College M. (2018). Whole mount in situ localization of miRNAs and mRNAs during somatic embryogenesis in Arabidopsis. Front. Plant Sci..

[32] Wójcik A.M., Gaj M.D. (2016). miR393 contributes to the embryogenic transition induced in vitro in Arabidopsis via the modification of the tissue sensitivity to auxin treatment. Planta.

[33] Long J.M., Liu C.Y., Feng M.Q., Liu Y., Wu X.M., Guo W.W. (2018). MiR156-SPL modules regulate induction of somatic embryogenesis in citrus callus. J. Exp. Bot..

[34] Szczygieł-Sommer A., Gaj M.D. (2019). The miR396–GRF regulatory module controls the embryogenic response in Arabidopsis via an auxin-related pathway. Int. J. Mol. Sci..

[35] Luján-Soto E., Dinkova T.D. (2021). Time to wake up: Epigenetic and small-RNA-mediated regulation during seed germination. Plants.

[36] Chuck G., Cigan A.M., Saeteurn K., Hake S. (2007). The heterochronic maize mutant Corngrass1 results from overexpression of a tandem microRNA. Nat. Genet..

[37] Xu M., Hu T., Zhao J., Park M.Y., Earley K.W., Wu G., Yang L., Poethig R.S. (2016). Developmental functions of miR156-regulated SQUAMOSA PROMOTER BINDING PROTEIN-LIKE (SPL) genes in *Arabidopsis thaliana*. PLoS Genet..

[38] Zhao L., Kim Y., Dinh T.T., Chen X. (2007). miR172 regulates stem cell fate and defines the inner boundary of APETALA3 and PISTILLATA expression domain in Arabidopsis floral meristems. Plant J..

[39] Tang J., Chu C. (2017). MicroRNAs in crop improvement: Fine-tuners for complex traits. Nat. Plants.

[40] Gasser C. (2015). Fruit development: miRNA pumps up the volume. Nat. Plants.

[41] José Ripoll J., Bailey L.J., Mai Q.-A., Wu S.L., Hon C.T., Chapman E.J., Ditta G.S., Estelle M., Yanofsky M.F. (2015). microRNA regulation of fruit growth. Nat. Plants.

[42] Liu P., Liu J., Dong H., Sun J. (2018). Functional regulation of Q by microRNA172 and transcriptional co-repressor TOPLESS in controlling bread wheat spikelet density. Plant Biotechnol. J..

[43] Zhang B., Chen X. (2021). Secrets of the MIR172 family in plant development and flowering unveiled. PLoS Biol..

[44] Lian H., Wang L., Ma N., Zhou C.M., Han L., Zhang T.Q., Wang J.W. (2021). Redundant and specific roles of individual MIR172 genes in plant development. PLoS Biol..

[45] Ó’Maoiléidigh D.S., van Driel A.D., Singh A., Sang Q., Le Bec N., Vincent C., de Olalla E.B.G., Vayssières A., Romera Branchat M., Severing E. (2021). Systematic analyses of the MIR172 family members of Arabidopsis define their distinct roles in regulation of APETALA2 during floral transition. PLoS Biol..

[46] Zhu Q.H., Helliwell C.A. (2011). Regulation of flowering time and floral patterning by miR172. J. Exp. Bot..

[47] Nakano T., Suzuki K., Fujimura T., Shinshi H. (2006). Genome-Wide Analysis of the ERF Gene Family in Arabidopsis and Rice. Plant Physiol..

[48] Ouakfaoui S.E., Schnell J., Abdeen A., Colville A., Labbé H., Han S., Baum B., Laberge S., Miki B. (2010). Control of somatic embryogenesis and embryo development by AP2 transcription factors. Plant Mol. Biol..

[49] Horstman A., Fukuoka H., Muino J.M., Nitsch L., Guo C., Passarinho P., Sanchez-Perez G., Immink R., Angenent G., Boutilier K. (2015). AIL and HDG proteins act antagonistically to control cell proliferation. Development.

[50] Mantiri F.R., Kurdyukov S., Lohar D.P., Sharopova N., Saeed N.A., Wang X.-D., Vandenbosch K.A., Rose R.J. (2008). The transcription factor MtSERF1 of the ERF subfamily identified by transcriptional profiling is required for somatic embryogenesis induced by auxin plus cytokinin in Medicago truncatula. Plant Physiol..

[51] Wu G., Park M.Y., Conway S.R., Wang J., Weigel D., Poethig R.S. (2009). The Sequential Action of miR156 and miR172 Regulates Developmental Timing in Arabidopsis. Cell.

[52] Zhang B., Wang L., Zeng L., Zhang C., Ma H. (2015). Arabidopsis TOE proteins convey a photoperiodic signal to antagonize CONSTANS and regulate flowering time. Genes Dev..

[53] Elliott R.C., Betzner A.S., Huttner E., Oakes M.P., Tucker W.Q.J., Gerentes D., Perez P., Smyth D.R. (1996). AINTEGUMENTA, an APETALA2-like gene of arabidopsis with pleiotropic roles in ovule development and floral organ growth. Plant Cell.

[54] Chuck G., Meeley R.B., Hake S. (1998). The control of maize spikelet meristem fate by the APETALA2-like gene indeterminate spikelet1. Genes Dev..

[55] Moose S.P., Sisco P.H. (1996). Glossy15, an APETALA2-like gene from maize that regulates leaf epidermal cell identity. Genes Dev..

[56] Adamczyk B.J., Lehti-shiu M.D., Fernandez D.E., Drive L. (2007). The MADS domain factors AGL15 and AGL18 act redundantly as repressors of the floral transition in Arabidopsis. Plant J..

[57] Würschum T., Groß-Hardt R., Laux T. (2006). APETALA2 regulates the stem cell niche in the Arabidopsis shoot meristem. Plant Cell.

[58] Plotnikova A., Kellner M.J., A. S.M., Mosiolek M., Nodine M.D. (2019). MicroRNA dynamics and functions during Arabidopsis embryogenesis. Plant Cell.

[59] Hofmann F., Schon M.A., Nodine M.D. (2019). The embryonic transcriptome of *Arabidopsis thaliana*. Plant Reprod..

[60] Kumar V., Thakur J.K., Prasad M. (2021). Histone acetylation dynamics regulating plant development and stress responses. Cell. Mol. Life Sci..

[61] Turner B.M. (2000). Histone acetylation and an epigenetic code. BioEssays.

[62] Feng W., Michaels S.D. (2015). Accessing the inaccessible: The organization, transcription, replication, and repair of heterochromatin in plants. Annu. Rev. Genet..

[63] Rodríguez-Sanz H., Manzanera J.A., Solís M.T., Gómez-Garay A., Pintos B., Risueño M.C., Testillano P.S. (2014). Early markers are present in both embryogenesis pathways from microspores and immature zygotic embryos in cork oak, *Quercus suber* L. BMC Plant Biol..

[64] Pérez M., Viejo M., LaCuesta M., Toorop P., Cañal M.J. (2015). Epigenetic and hormonal profile during maturation of Quercus Suber L. somatic embryos. J. Plant Physiol..

[65] Yakovlev I.A., Carneros E., Lee Y.K., Olsen J.E., Fossdal C.G. (2016). Transcriptional profiling of epigenetic regulators in somatic embryos during temperature induced formation of an epigenetic memory in Norway spruce. Planta.

[66] Tanaka M., Kikuchi A., Kamada H. (2008). The arabidopsis histone deacetylases HDA6 and HDA19 contribute to the repression of embryonic properties after germination. Plant Physiol..

[67] Wójcikowska B., Botor M., Morończyk J., Wójcik A.M., Nodzyński T., Karcz J., Gaj M.D. (2018). Trichostatin a triggers an embryogenic transition in Arabidopsis explants via an auxin-related pathway. Front. Plant Sci..

[68] Uddenberg D., Valladares S., Abrahamsson M., Sundström J.F., Sundås-Larsson A., von Arnold S. (2011). Embryogenic potential and expression of embryogenesis-related genes in conifers are affected by treatment with a histone deacetylase inhibitor. Planta.

[69] Abrahamsson M., Valladares S., Merino I., Larsson E., von Arnold S. (2017). Degeneration pattern in somatic embryos of *Pinus sylvestris* L. Vitr. Cell. Dev. Biol.-Plant.

[70] Wang Z., Cao H., Chen F., Liu Y. (2014). The roles of histone acetylation in seed performance and plant development. Plant Physiol. Biochem..

[71] Nowak K., Morończyk J., Wójcik A., Gaj M.D. (2020). AGL15 controls the embryogenic reprogramming of somatic cells in Arabidopsis through the histone acetylation-mediated repression of the mirna biogenesis genes. Int. J. Mol. Sci..

[72] Todesco M., Rubio-Somoza I., Paz-Ares J., Weigel D. (2010). A collection of target mimics for comprehensive analysis of MicroRNA function in *Arabidopsis thaliana*. PLoS Genet..

[73] Coego A., Brizuela E., Castillejo P., Ruíz S., Koncz C., del Pozo J.C., Piñeiro M., Jarillo J.A., Paz-Ares J., León J. (2014). The TRANSPLANTA collection of Arabidopsis lines: A resource for functional analysis of transcription factors based on their conditional overexpression. Plant J..

[74] Nodine M.D., Bartel D.P. (2010). MicroRNAs prevent precocious gene expression and enable pattern formation during plant embryogenesis. Genes Dev..

[75] Gaj M.D. (2001). Direct somatic embryogenesis as a rapid and efficient system for in vitro regeneration of *Arabidopsis thaliana*. Plant Cell. Tissue Organ Cult..

[76] Gamborg O.L., Miller R.A., Ojima K. (1968). Nutrient requirements of suspension cultures of soybean root cells. Exp. Cell Res..

[77] Speth C., Laubinger S., Staiger D. (2014). Rapid and Parallel Quantification of Small and Large RNA Species. Plant Circadian Networks: Methods and Protocols.

[78] Yelagandula R., Osakabe A., Axelsson E., Berger F., Kawashima T., Busch W. (2017). Genome-Wide Profiling of Histone Modifications and Histone Variants in *Arabidopsis thaliana* and *Marchantia polymorpha*. Plant Genomics: Methods and Protocols.

[79] Wu G., Poethig R.S. (2006). Temporal regulation of shoot development in *Arabidopsis thaliana* by miRr156 and its target SPL3. Development.

[80] Yant L., Mathieu J., Dinh T.T., Ott F., Lanz C., Wollmann H., Chen X., Schmid M. (2010). Orchestration of the floral transition and floral development in arabidopsis by the bifunctional transcription factor APETALA2. Plant Cell.

[81] Causier B., Ashworth M., Guo W., Davies B. (2012). The TOPLESS interactome: A framework for gene repression in Arabidopsis. Plant Physiol..

[82] Krogan N.T., Hogan K., Long J.A. (2012). APETALA2 negatively regulates multiple floral organ identity genes in Arabidopsis by recruiting the co-repressor TOPLESS and the histone deacetylase HDA19. Development.

[83] Huijser P., Schmid M. (2011). The control of developmental phase transitions in plants. Development.

[84] Li T., Chen J., Qiu S., Zhang Y., Wang P., Yang L., Lu Y., Shi J. (2012). Deep sequencing and microarray hybridization identify conserved and species-specific microRNAs during somatic embryogenesis in hybrid yellow poplar. PLoS ONE.

[85] Rodrigues A.S., Chaves I., Costa B.V., Lin Y.C., Lopes S., Milhinhos A., Van de Peer Y., Miguel C.M. (2019). Small RNA profiling in Pinus pinaster reveals the transcriptome of developing seeds and highlights differences between zygotic and somatic embryos. Sci. Rep..

[86] Chen Y., Li X., Su L., Chen X., Zhang S., Xu X., Zhang Z., Chen Y., Xuhan X., Lin Y. (2018). Genome-wide identification and characterization of long non-coding RNAs involved in the early somatic embryogenesis in Dimocarpus longan Lour. BMC Genomics.

[87] Wójcikowska B., Jaskóła K., Gasiorek P., Meus M., Nowak K., Gaj M.D. (2013). LEAFY COTYLEDON2 (LEC2) promotes embryogenic induction in somatic tissues of Arabidopsis, via YUCCA-mediated auxin biosynthesis. Planta.

[88] Wójcikowska B., Gaj M.D. (2017). Expression profiling of AUXIN RESPONSE FACTOR genes during somatic embryogenesis induction in Arabidopsis. Plant Cell Rep..

[89] Schwab R., Palatnik J.F., Riester M., Schommer C., Schmid M., Weigel D. (2005). Specific effects of microRNAs on the plant transcriptome. Dev. Cell.

[90] Aukerman M.J., Sakai H. (2003). Regulation of flowering time and floral organ identity by a microRNA and its Apetala2-like target genes. Plant Cell.

[91] Kasschau K.D., Xie Z., Allen E., Llave C., Chapman E.J., Krizan K.A., Carrington J.C. (2003). P1/HC-Pro, a viral suppressor of RNA silencing, interferes with Arabidopsis development and miRNA function. Dev. Cell.

[92] Chen X. (2004). A MicroRNA as a translational repressor of APETALA2 in Arabidopsis flower development. Science.

[93] Wollmann H., Mica E., Todesco M., Long J.A., Weigel D. (2010). On reconciling the interactions between APETALA2, miR172 and AGAMOUS with the ABC model of flower development. Development.

[94] Grigorova B., Mara C., Hollender C., Sijacic P., Chen X., Liu Z. (2011). LEUNIG and SEUSS co-repressors regulate miR172 expression in arabidopsis flowers. Development.

[95] Su Y.H., Zhao X.Y., Liu Y.B., Zhang C.L., O’Neill S.D., Zhang X.S. (2009). Auxin-induced WUS expression is essential for embryonic stem cell renewal during somatic embryogenesis in Arabidopsis. Plant J..

[96] Zheng W., Zhang X., Yang Z., Wu J., Li F., Duan L., Liu C., Lu L., Zhang C., Li F. (2014). AtWuschel promotes formation of the embryogenic callus in Gossypium hirsutum. PLoS ONE.

[97] Krogan N.T., Long J.A. (2009). Why so repressed? Turning off transcription during plant growth and development. Curr. Opin. Plant Biol..

[98] Mozgová I., Muñoz-Viana R., Hennig L. (2017). PRC2 represses hormone-induced somatic embryogenesis in vegetative tissue of *Arabidopsis thaliana*. PLoS Genet..

[99] Liu X., Yang S., Zhao M., Luo M., Yu C.W., Chen C.Y., Tai R., Wu K. (2014). Transcriptional repression by histone deacetylases in plants. Mol. Plant.

[100] Rensing S.A., Lang D., Schumann E., Reski R., Hohe A. (2005). EST sequencing from embryogenic Cyclamen persicum cell cultures identifies a high proportion of transcripts homologous to plant genes involved in somatic embryogenesis. J. Plant Growth Regul..

[101] Yang X., Li L. (2012). Analyzing the microRNA Transcriptome in Plants Using Deep Sequencing Data. Biology.

[102] 1Zheng C., Ye M., Sang M., Wu R. (2019). A regulatory network for mir156-SPL module in *Arabidopsis thaliana*. Int. J. Mol. Sci..

[103] Fornara F., Coupland G. (2009). Plant phase transitions make a SPLash. Cell.

[104] Yu N., Cai W.J., Wang S., Shan C.M., Wang L.J., Chena X.Y. (2010). Temporal control of trichome distribution by microRNA156-targeted SPL genes in *Arabidopsis thaliana*. Plant Cell.

[105] Rubio-Somoza I., Zhou C.M., Confraria A., Martinho C., Von Born P., Baena-Gonzalez E., Wang J.W., Weigel D. (2014). Temporal control of leaf complexity by miRNA-regulated licensing of protein complexes. Curr. Biol..

[106] Zhang Q.Q., Wang J.G., Wang L.Y., Wang J.F., Wang Q., Yu P., Bai M.Y., Fan M. (2020). Gibberellin repression of axillary bud formation in Arabidopsis by modulation of DELLA-SPL9 complex activity. J. Integr. Plant Biol..

[107] Ohta M., Matsui K., Hiratsu K., Shinshi H., Ohme-Takagi M. (2001). Repression domains of class II ERF transcriptional repressors share an essential motif for active repression. Plant Cell.

[108] Kagale S., Rozwadowski K. (2011). EAR motif-mediated transcriptional repression in plants: An underlying mechanism for epigenetic regulation of gene expression. Epigenetics.

[109] Yang J., Liu Y., Yan H., Tian T., You Q., Zhang L., Xu W., Su Z. (2018). PlantEAR: Functional analysis platform for plant EAR motif-containing proteins. Front. Genet..

[110] Yu S., Galvão V.C., Zhang Y.C., Horrer D., Zhang T.Q., Hao Y.H., Feng Y.Q., Wang S., Schmid M., Wang J.W. (2012). Gibberellin regulates the Arabidopsis floral transition through miR156-targeted SQUAMOSA PROMOTER BINDING-LIKE transcription factors. Plant Cell.

[111] Xie Y., Liu Y., Ma M., Zhou Q., Zhao Y., Zhao B., Wang B., Wei H., Wang H. (2020). Arabidopsis FHY3 and FAR1 integrate light and strigolactone signaling to regulate branching. Nat. Commun..

[112] Jiménez V.M. (2005). Involvement of plant hormones and plant growth regulators on in vitro somatic embryogenesis. Plant Growth Regul..

[113] Fouracre J.P., Scott Poethig R. (2019). Role for the shoot apical meristem in the specification of juvenile leaf identity in Arabidopsis. Proc. Natl. Acad. Sci. USA.

[114] Sun Z., Su C., Yun J., Jiang Q., Wang L., Wang Y., Cao D., Zhao F., Zhao Q., Zhang M. (2019). Genetic improvement of the shoot architecture and yield in soya bean plants via the manipulation of GmmiR156b. Plant Biotechnol. J..

[115] Teotia S., Tang G. (2015). To bloom or not to bloom: Role of micrornas in plant flowering. Mol. Plant.

[116] Thakare D., Tang W., Hill K., Perry S.E. (2008). The MADS-domain transcriptional regulator AGAMOUS-LIKE15 promotes somatic embryo development in Arabidopsis and soybean. Plant Physiol..

[117] Zheng Q., Zheng Y., Perry S.E. (2013). AGAMOUS-Like15 promotes somatic embryogenesis in Arabidopsis and soybean in part by the control of ethylene biosynthesis and response. Plant Physiol..

[118] Zheng Q., Perry S.E. (2014). Alterations in the transcriptome of soybean in response to enhanced somatic embryogenesis promoted by orthologs of Agamous-like15 and Agamous-like18. Plant Physiol..

[119] Wang L., Zhou C., Mai Y.-X., Li L.-Z., Gao J., Shang G.-D., Lian H., Han L., Zhang T., Tang H.-B. (2019). A spatiotemporally regulated transcriptional complex underlies heteroblastic development of leaf hairs in *Arabidopsis thaliana*. EMBO J..

[120] Chandler J.W. (2008). Cotyledon organogenesis. J. Exp. Bot..

[121] Kurczyńska E.U., Gaj M.D., Ujczak A., Mazur E. (2007). Histological analysis of direct somatic embryogenesis in *Arabidopsis thaliana* (L.) Heynh. Planta.

[122] Karami O., Philipsen C., Rahimi A., Nurillah A.R., Boutilier K., Offringa R. (2021). Endogenous auxin directs development of embryonic stem cells into somatic proembryos in *Arabidopsis*. bioRxiv.

[123] Tameshige T., Fujita H., Watanabe K., Toyokura K., Kondo M., Tatematsu K., Matsumoto N., Tsugeki R., Kawaguchi M., Nishimura M. (2013). Pattern dynamics in adaxial-abaxial specific gene expression are modulated by a plastid retrograde signal during *Arabidopsis thaliana* leaf development. PLoS Genet..

[124] Huang T., Harrar Y., Lin C., Reinhart B., Newell N.R., Talavera-Rauh F., Hokin S.A., Kathryn Barton M., Kerstetter R.A. (2014). Arabidopsis KANADI1 acts as a transcriptional repressor by interacting with a specific cis-element and regulates auxin biosynthesis, transport, and signaling in opposition to HD-ZIPIII factors. Plant Cell.

[125] Bianchi M., Renzini A., Adamo S., Moresi V. (2017). Coordinated actions of microRNAs with other epigenetic factors regulate skeletal muscle development and adaptation. Int. J. Mol. Sci..

[126] Xu Y., Zhang L., Wu G. (2018). Epigenetic regulation of juvenile-to-adult transition in plants. Front. Plant Sci..

[127] Horstman A., Bemer M., Boutilier K. (2017). A transcriptional view on somatic embryogenesis. Regeneration.

[128] Yuan L., Song X., Zhang L., Yu Y., Liang Z., Lei Y., Ruan J., Tan B., Liu J., Li C. (2021). The transcriptional repressors VAL1 and VAL2 recruit PRC2 for genome-wide Polycomb silencing in Arabidopsis. Nucleic Acids Res..

[129] Baile F., Merini W., Hidalgo I., Calonje M. (2021). EAR domain-containing transcription factors trigger PRC2-mediated chromatin marking in Arabidopsis. Plant Cell.

