# Supplementary material for: miR172 Regulates WUS during Somatic Embryogenesis in Arabidopsis via AP2
Source: Cells. 2022 Feb 17;11(4):718. doi: 10.3390/cells11040718 (PMC8869827; doi:10.3390/cells11040718)
Supplement: Supplementary file 1 [file cells-11-00718-s001.zip › cells-1536854-supplementary.pdf]

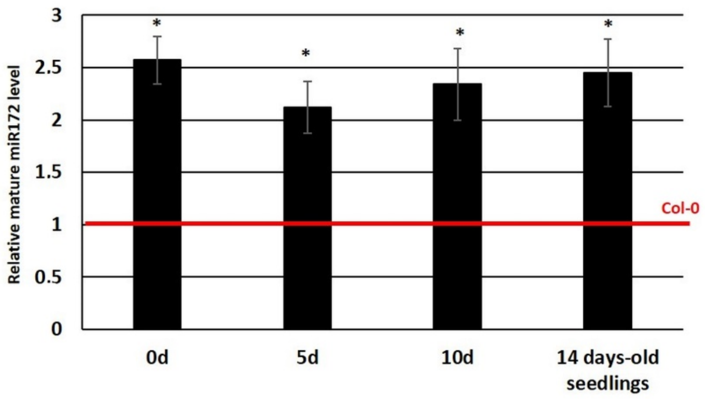

Figure S1. Level of mature miR172 in the embryogenic culture and 14-day-old seedlings of the 35S::*MIR172D* line. The relative miRNA level was normalised to an internal control (*At4g27090*) and calibrated to the WT (Col-0) culture of the same age (0d, 5d, and 10d) and 14-day-old seedlings. * – value significantly different from the Col-0 culture of the same age (P<0.05; n = 3 ± SD) and 14-day-old seedlings.

Table S1: List of the primers that were used for the Real Time qPCR analysis.

Gene	Primers sequence
TOE1	pF - AATAATCCCGCCGAGGGAAGAG<br>pR - AACAATGGTGGTGGTTGTGGTC
TOE2	pF - TGGAGCAGCTTCATGGAAACATGG<br>pR - GCTTCCCTTCCCTTCCATTGTACG
TOE3	pF - CCGATAAAGAAGAGCCGACGTG<br>pR - TGTGCAGTGTCAAATCCACCTAAG
SMZ	pF – AAGGAGCCATGAAGTTTGGTG<br>pR - TGCTGATAAAAGGGAATCCTG
SNZ	pF – AATGAGCACAACGATCTCGAG<br>pR - GCCCATAGTAAAGGAAATGAG
AP2	pF – ATACTCCCAATTCAAACCACC<br>pR - TCAAGAAGGTCTCATGAGAGG
WUS	pF – TCACCATCATCACGGTGTTC<br>pR - AGAACAGTCTTGTTCCATAGA
AGL15	pF - CAAGGGCTTGAATCCTCTGA<br>pR- GTTGTTCCTTGAGGCGTGAT
miR712	pR – CGGGCGGAGAATCTTGATGATG<br>pR - GTGCAGGGTCCGAGGT
WUS TSS+300	pF- GCCACAGCATCAGCATCATC<br>pR- CGACACGTGTAACCACCAGA
WUS 3′UTR	pF – CATCATAGAGATAAAACGGTTGTCA<br>pR - TCGCTCGGGATTAAAGCTC

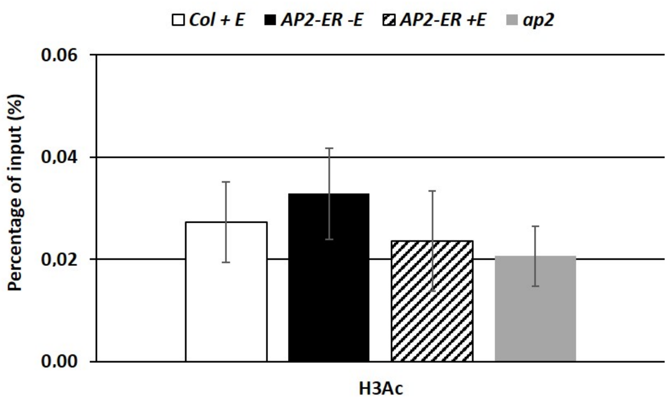

Figure S2. H3ac enrichment in the chromatin fragment that was bound to the 3'UTR of the *WUS* gene in the explants of Col-0 (WT), 35S::*AP2*-ER and *ap2*. *AP2* overexpression was induced with β-estradiol (+E). A statistical analysis indicated no differences between the compared combinations ($P<0.05$; n = 3 ± SD).

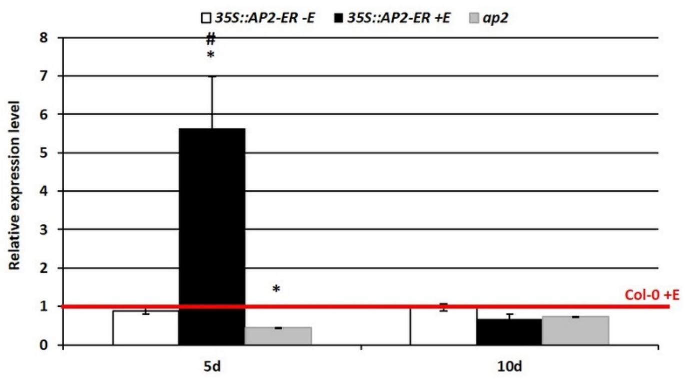

Figure S3: Expression analysis of *AGL15* in the SE culture in *AP2*-induced overexpressor line and the *ap2* mutant. The relative transcript level was normalised to an internal control (*At4g27090*) and calibrated to the WT (Col-0) culture of the same age. * values significantly different from the Col-0 culture of the same age (P<0.05; n = 3 ± SD); # - values significantly different from the 35S::*AP2*-ER -E culture of the same age (P<0.05; n = 3 ± SD).
